# Design of a Low-Cost Indoor Navigation System for Food Delivery Robot Based on Multi-Sensor Information Fusion

**DOI:** 10.3390/s19224980

**Published:** 2019-11-15

**Authors:** Yunlong Sun, Lianwu Guan, Zhanyuan Chang, Chuanjiang Li, Yanbin Gao

**Affiliations:** 1 College of Automation, Harbin Engineering University, Harbin 150001, China; sunyunlong@hrbeu.edu.cn (Y.S.); gaoyanbin@hrbeu.edu.cn (Y.G.); 2 College of Information, Mechanical and Electrical Engineering, Shanghai Normal University, Shanghai 200234, China; licj@shnu.edu.cn

**Keywords:** wheeled robot, robot control, odometer positioning method, ultra-wide band localization, extended Kalman filter

## Abstract

As the restaurant industry is facing labor shortage issues, the use of meal delivery robots instead of waiters/waitresses not only allows the customers to experience the impact of robot technology but also benefits the restaurant business financially by reducing labor costs. Most existing meal delivery robots employ magnetic navigation technologies, which require magnetic strip installation and changes to the restaurant decor. Once the moving path is changed, the magnetic strips need to be re-laid. This study proposes multisource information fusion, i.e., the fusion of ultra-wide band positioning technology with an odometer and a low-cost gyroscope accelerometer, to achieve the positioning of a non-rail meal delivery robot with navigation. By using a low-cost electronic compass and gyroscope accelerometer, the delivery robot can move along a fixed orbit in a flexible and cost-effective manner with steering control. Ultra-wide band (UWB) and track estimation algorithm are combined by extended Kalman filter (EKF), and the positioning error after fusion is about 15 cm, which is accepted by restaurants. In summary, the proposed approach has some potential for commercial applications.

## 1. Introduction

Food and beverage marketing statistics have shown that the value of the Chinese food industry reached 2T yuan in 2015. However, with rising labor costs, the profit margins of the food and beverage industry have remained low. In China, the gradual increase in labor costs has led to labor shortages, contributing toward the replacement of humans with robots. Such a policy not only reduces labor costs but also improves efficiency and increases corporate earnings [1]. For example, at restaurants, delivery robots not only reduce labor costs but also increase the automatic control performance and practicality of the robots. Compared to industrial robots, service robots are used in social settings, hence, their precision and safety requirements are higher. Most service robots are designed for a particular environment to assist humans in accomplishing some specific tasks, e.g., restaurant diners.

The vast majority of commercial delivery robots are controlled by magnetic induction or optical tracking. Such robots consist of a head unit, trunk assembly, and robotic arm. The literature [2] proposed a daily life assistance robot with projection function. The literature [3] designed a front-desk service robot named “Black Bot” with voice control function. The robotic arm usually holds an object and its main body moves along a guide rail. The main body consists of a controller, a battery, motor drives, and a voice interface. The guide rail with magnetic strips can reflect photoelectric beams. Positioning and obstacle avoidance are achieved by a tracking device in the body. The disadvantages of this approach are as follows. Poor environmental adaptability: In a barrier-free environment in the laboratory, the robot performs well. However, in an actual restaurant, its anti-interference ability is poor [4]. Moreover, it is vulnerable to light and surface stains. Such external interference can severely affect the operation of the robot. High installation and maintenance costs: Current delivery robots work only in accordance with a preset trajectory, which has to be installed along a track on the ground. This will not only affect the layout and appearance of the restaurant but also increase installation costs. Inflexible service schedule: If the moving schedule is changed, the path needs to be re-laid, which is usually infeasible.

Indoor location-based services are challenging owing to the vast coverage required and the scalability of positioning systems [5]. At present, the mainstream interior positioning technologies include magnetic navigation, simultaneous localization and mapping (SLAM) [6,7], Wi-Fi positioning, radio frequency identification (RFID) positioning, and UWB positioning [8,9]. There have been many studies on Wi-Fi indoor positioning technology. Xujian and Hao [10] proposed an improved Kalman-filtering-based WiFi indoor positioning algorithm. Zhang et al. [11] presented a domain-clustering-based WiFi indoor positioning algorithm. In UWB field, Ubisense Co. Ltd. from the UK has produced the UbisB 7000 positioning system based on UWB technology, which uses Time Difference of Arrival (TDOA) and Activity on Arrow (AOA) hybrid positioning algorithm to achieve 15 cm accuracy in typical applications. AETHER WIRE & LOCATION Co. Ltd. of the United States introduced the localizers system with a range accuracy of 1cm and a node volume of 8 mm [12]. Their common shortcoming is that they are expensive. When these indoor positioning technologies are used independently, they tend to have poor positioning accuracy and low reliability. In addition, they are time-consuming and entail high costs. Although guiding a robot using magnetic navigation as a means of positioning does not appear to involve positioning errors, the robot stops when it encounters obstacles and it cannot make an autonomous detour. In addition, when an out-of-orbit accident occurs, the operation has to be resumed by the staff, and the flexibility is low. Another common method for robot positioning is based on SLAM, which allows independent navigation, obstacle avoidance, and path planning. Compared with magnetic navigation, the flexibility of the feeding robot is improved considerably. However, when faced with many obstacles or surrounded by humans, the radar cannot scan the surrounding environment sufficiently, resulting in a loss of coordinates. Wi-Fi [13] and RFID technologies cannot meet the precision requirements of indoor positioning for meal delivery robots. To improve accuracy, researchers have proposed the use of UWB technology. UWB signals have the advantages of high penetration, high multi-path resolution, and low transmitting power. UWB positioning can achieve centimeter-scale location accuracy without accumulating errors [14,15]. Considering the environment in a restaurant, the use of only UWB technology results in low positioning accuracy, e.g., around 30–40 cm. In this situation, using an inertial measurement unit (IMU) is a good choice. The Inertial Navigation System (INS) has good short-term accuracy and does not rely on any external sources for determining position and attitude. Guan et al. [16] presented a low-cost, high-integrated, and small-size mems inertial navigation system. Combining an IMU with UWB technology can improve the positioning accuracy [17,18]. However, the stability is poor while turning or changing speed and the overall accuracy of the system is thus reduced [19,20]. In addition to the fusion of UWB and IMU technologies, visual odometer correction can improve the positioning accuracy. However, it increases the hardware cost. Moreover, the installation is complex, and the system is sensitive to light [21]. Moreover, a large number of network nodes are required to complete a wide range of indoor target tracking tasks, which is bound to introduce concerns related to network structure optimization as well as multi-node/multi-cluster coordination and communication. Among navigation technologies without beacons, the most commonly used method is the analysis of the pedestrian trajectory by an inertial navigation system [22]. To overcome the shortcomings of a single technology for interior positioning, it is necessary to integrate such technology with multi-sensor information to realize a high-precision, high-reliability, and low-cost positioning system.

This paper presents a meal delivery robot with positioning and navigation control based on the fusion of information from multiple sources or technologies, namely UWB positioning, an odometer, a low-cost gyroscope accelerometer, and an electronic compass. We establish the kinematics model of the delivery robot. To improve the positioning accuracy and stability effectively, we fuse the positioning results of the UWB system with those of odometer and a dead reckoning algorithm. The algorithm used for the fusion is an extended Kalman filter (EKF) fusion algorithm, which is suitable for discrete systems in the presence of Gaussian white noise. The designed robot can move along the preset path without the need for laying a navigation track. Moreover, when the restaurant changes its layout, we need to modify only the default track in the software.

This paper is organized as follows: Section 1 introduces the current situation of service robots at home and abroad and extends to the food delivery machine. In addition, the research status of indoor positioning technology is expounded, and the advantages and disadvantages of various positioning technologies are roughly compared. At the same time, the main multi-sensor information fusion algorithms at home and abroad are introduced. Section 2 introduces the positioning system design, which including the robot modeling, odometer positioning method, and optimization, UWB positioning method and EKF fusion of the previous two algorithms. At last, meal delivery robot trajectory control is introduced. Section 3 introduces the experiment and result analysis. The experiment is divided into three parts: The UWB positioning system experiment, coordinate calculation experiment for improved odometer positioning method, and the EKF fusion algorithm coordinate fusion experiment. The previous two experiments obtained the positioning results of the UWB positioning system and the improved odometer positioning method, respectively, and analysis of the error and source. Finally, the EKF fusion algorithm is used to fuse the positioning results of the two algorithms to obtain better positioning results with better accuracy and stability. Section 4 is the conclusion. By this algorithm, the trajectory error of the food delivery robot can be less than 15 cm, whose precision is acceptable for the application field such as in a restaurant.

## 2. Positioning System Design

The delivery robot control system consists of a control panel, motors and motor drives, power supplies, UWB positioning systems, an infrared and ultrasonic sensor module, and a gyroscope attitude sensor.

The robot has three wheels. Two of the three wheels are motor-driven, while the third is a follower. Each motor-driven wheel is connected to a 90-W servo motor via a gearbox. Each motor drive is associated with an encoder data output. To realize autonomous navigation control of the robot, the control panel employs a STM32 [23] system to receive data from the infrared sensor, ultrasonic sensor, gyroscope accelerometer, and electronic compass in order to avoid obstacles, transmit information through the server (host computer), and control the motors according to the positioning information. The system also employs a low-cost IMU (9DoF-RazorIMU) [24], which provides the heading angle for robot steering.

**Figure 1:**
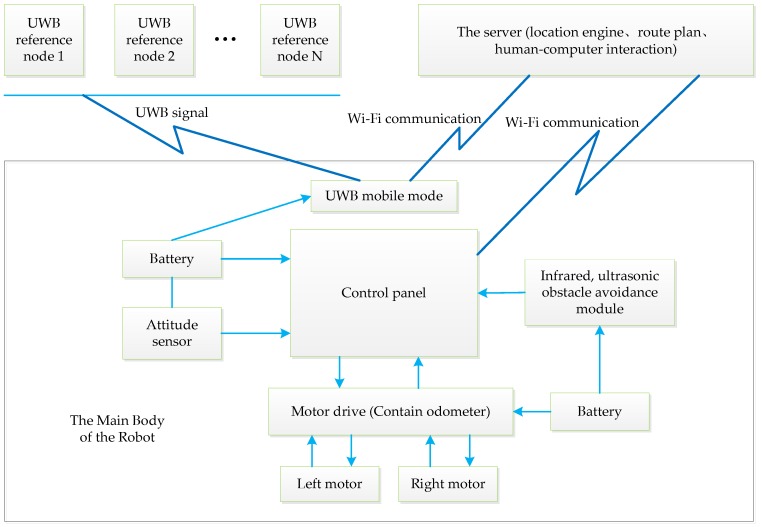
Structure diagram of the robot system.

As shown in Figure 1, the server calculates the robot’s current location in accordance with the UWB signal and then calculates the direction and speed of the robot according to the user-preset target point. The embedded firmware in the main control board modifies the speed of the left and right wheels to steer the robot, while the electronic compass provides angle feedback to achieve more precise steering control. Furthermore, the embedded firmware acquires signals from the obstacle avoidance sensor in real time. Thus, the robot operates an emergency brake within a certain distance from the obstacles. Obstacle avoidance is set as the highest priority to ensure the safe operation of the robot.

The meal delivery robot positioning system consists of a combination of UWB positioning and odometer positioning systems.

### 2.1. Traditional Odometer Positioning Method

The odometer signals are provided by the left and right motors, i.e., the odometer records the number of motor rotations and then performs calculations based on the real-time location. This is a low-cost design.

**Figure 2:**
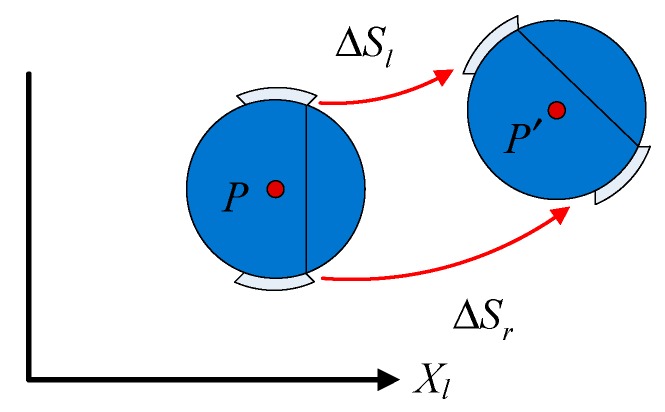
Movement track diagram of the robot in time *t*.

It is assumed that the trajectory of the food delivery robot is a segment of an arc, and if the rotation speed of the food delivery robot is zero, which means that the speeds of the left and right wheels are equal, the trajectory of movement of the robot is a straight line. Assume that the two-wheel distance of the food delivery robot is 2L and the center of its movement mechanism (the center of the two rounds of connection) is point p. After a certian time, the robot moves to point p’, and the arc lengths of the left and right wheels increase, as shown in Figure 2.

**Figure 3:**
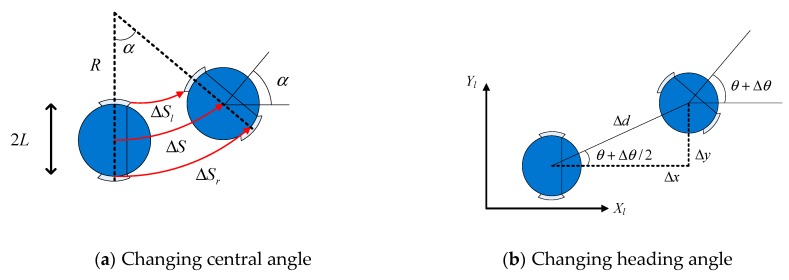
Changing angle of the robot in time *t*.

If the extension lines of the two wheels when the robot is at points p and p’ intersect, their intersection point is the center of the circle where the delivery robot’s movement trajectory is located. The radius of the circle is R, the actual walking arc length is ΔS, the left wheel moving arc length is $\Delta S_{l}$, the left wheel moving arc length is $\Delta S_{r}$ and the changing angle is α. Figure 3a shows the changing central angle of the robot in time *t*. Figure 3b shows the changing heading angle of the robot in time *t*.

The following formula can be obtained by arc length calculation:

(1) $$\Delta S_{l}=R\alpha ,\Delta S_{r}=(R+2L)\alpha$$

(2) $$\Delta S=(R+L)\alpha =\frac{\left(\Delta S_{l}+\Delta S_{r}\right)}{2}$$

Equation (1) can be rewritten as Equation (2):

(3) $$R=\frac{2L\Delta S_{l}}{\Delta S_{r}-\Delta S_{l}}$$

Thus, Equation (3) can be obtained:

(4) $$\Delta \theta =\alpha =\frac{\Delta S_{l}}{R}=\frac{\Delta S_{l}-\Delta S_{r}}{2L}$$

where Δθ is the changing angle of the delivery robot.

When the distance traveled by the delivery robot is extremely small, the incremental arc length ΔS is equivalent to a small straight line of length Δd. The following formula can be deduced from Figure 3b.

(5) $$\Delta x=\Delta d\cos(\theta +\frac{\Delta \theta }{2})$$

(6) $$\Delta y=\Delta d\sin(\theta +\frac{\Delta \theta }{2})$$

The final coordinates *x*, *y* and the heading angle θ can be expressed as follows:

(7) $$x=x+\Delta x=x+\Delta d\cos(\theta +\frac{\Delta \theta }{2})$$

(8) $$y=y+\Delta y=y+\Delta d\sin(\theta +\frac{\Delta \theta }{2})$$

(9) $$\theta =\theta +\Delta \theta =\theta +\frac{\Delta S_{l}+\Delta S_{r}}{2L}$$

From the analysis presented above, if the delivery robot is described by the function $f(x,y,\theta ,\Delta S_{l},\Delta S_{r})$ at point p’, the following equation can be obtained:

(10) $$p^{\prime }=f(x,y,\theta ,\Delta S_{l},\Delta S_{r})=\left(\begin{array}{c} x\\ y\\ \theta \end{array}\right)+\left(\begin{array}{l} x+\frac{\Delta S_{l}+\Delta S_{r}}{2}\cos(\theta +\frac{\Delta S_{l}-\Delta S_{r}}{4L})\\ y+\frac{\Delta S_{l}+\Delta S_{r}}{2}\sin(\theta +\frac{\Delta S_{l}-\Delta S_{r}}{4L})\\ \theta +\frac{\Delta S_{r}+\Delta S_{l}}{2L} \end{array}\right)$$

### 2.2. Improved Odometer Positioning Method

To address the accumulated error of the traditional odometer positioning method and inability of the system to accurately reflect the coordinates and heading angle of the delivery robot under slippery and low-friction conditions, an improved odometer positioning method is adopted, which uses an external sensor to measure the heading angle and angle change during the sampling period on the basis of photoelectric signals.

This method uses the heading angle α measured by an IMU, because the heading angle calculated by a photoelectric odometer cannot accurately reflect the heading angle of the delivery robot. However, considering that MEMS gyro sensors and magnetic sensors in the IMU are susceptible to zero-point drift, white noise, temperature, acceleration, integral errors, and changing magnetic fields, the error in the data over a short period of time may be relatively large. If the data in the sampling period interval fluctuates considerably, the change in the heading angle will be significant, and the heading angle for the food delivery robot will not be accurate. Therefore, the heading angle change Δθ calculated by the photoelectric odometer is still used in the improved odometer positioning method. The formulas of the improved odometer positioning method formulas are as follows, where LocationX and LocationY are the coordinates of the delivery robot [25].

(11) $$\Delta X=V_{R\mathrm{ob}}\times \Delta t\times \cos\left(\alpha +\frac{\Delta \theta }{2}\right)$$

(12) $$\Delta Y=V_{R\mathrm{ob}}\times \Delta t\times \sin\left(\alpha +\frac{\Delta \theta }{2}\right)$$

(13) LocationX=LocationX+ΔX

(14) LocationY=LocationY+ΔY

**Figure 4:**
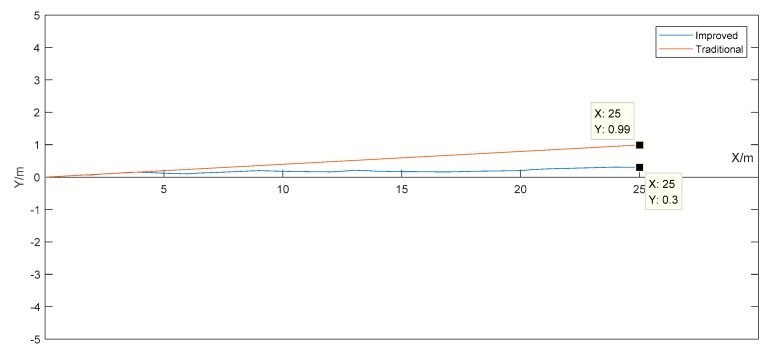
Delivery robot motion trajectory simulation diagram.

The simulation experiment assumes that the delivery robot moves from the origin in the two-dimensional *X*-*Y* coordinate system, i.e., (0,0) by 25 m along the *X*-axis direction. The initial direction of the IMU is 0°, and its noise is assumed to be Gaussian white noise. Factors such as magnetic field interference in the environment are not considered temporarily. Figure 4 shows a simulation diagram of the delivery robot motion trajectories obtained by the traditional odometer positioning method and the improved odometer positioning method in the two-dimensional plane.

The coordinates calculated by both the traditional odometer positioning method and the improved odometer positioning method include errors. However, the error in the latter is obviously smaller than that in the former. The error is mainly caused by the heading angle change within the sampling period. It is analyzed as follows.

Assume that the sampling interval of the master control system is Δt, the distance traveled by the delivery robot is $\Delta S=V_{Rob}\times \Delta t$, and the change in the heading angle is Δθ. If the delivery robot moves from the origin (0,0) by 1 m to the coordinate point (1,0), the theoretical displacement S is 1 m and the actual displacement ΔS is $1+e_{s}$ m. The theoretical heading angle variation is 0° and the actual heading angle variation Δθ is $0+e_{s}$°, where $e_{s}$ and $e_{\theta }$ are the errors in the distance and heading angle variation, respectively.

Thus, we have

(15) $$\begin{array}{l} \Delta X=V_{Rob}\times \Delta t\times \cos\left(\theta +\frac{\theta }{2}\right)=\Delta S\times \cos\left(\theta +\frac{\Delta \theta }{2}\right)=\left(1+e_{s}\right)\cos\left(0+\frac{0+e_{\theta }}{2}\right)\\ \Delta Y=V_{Rob}\times \Delta t\times \sin\left(\theta +\frac{\theta }{2}\right)=\Delta S\times \sin\left(\theta +\frac{\Delta \theta }{2}\right)=\left(1+e_{s}\right)\sin\left(0+\frac{0+e_{\theta }}{2}\right) \end{array}$$

For example, suppose that $e_{s}$ is 0.001 m and $e_{\theta }$ is 0°. Then, Δx and Δy are given by

$$\begin{array}{l} \Delta x=(1.001)\times \cos(0)=1.001\\ \Delta y=(1.001)\times \sin(0)=0 \end{array}$$

It can be seen that $e_{s}$ affects only the direction of the movement of the robot.

If we assume that $e_{s}=0m$ and $e_{\theta }=2^{\circ }$, then Δx and Δy are given by

$$\begin{array}{l} \Delta x=\cos(1)\approx 0.998\\ \Delta y=\sin(1)\approx 0.0175 \end{array}$$

It can be seen that $e_{\theta }$ affects both the *X*-axis and the *Y*-axis values. With time, the error along the two axes will be introduced once in each interval Δt, which will contribute to the cumulative error of the final positioning result. Thus, the cumulative error will increase with time. The longer the running time and the longer the operating distance, the larger is the error in the positioning coordinates.

The traditional odometer positioning method has high accuracy and good stability for a short duration and short distance. However, for a long duration and long distance, it cannot accurately reflect the coordinates of the delivery robot. The improved odometer positioning method based on the IMU is less stable than the traditional odometer positioning method, but it can reflect the coordinates of the delivery robot accurately over a long duration and long distance.

### 2.3. UWB Positioning Method

As shown in Figure 4, the UWB positioning system builds a reference on the basis of the fixed reference nodes in the four corners of the room (anchors) and a mobile node (node labels) mounted on the robot. UWB communication between the mobile node and the fixed nodes is achieved via wireless data transmission [17]. The UWB positioning algorithm is implemented by the embedded firmware.

The fixed nodes are installed on the ceiling of the dining room to reduce isolation. The bi-directional ranging method is used to convert the distance $\left(l_{1},l_{2},l_{3}\right)$ between the fixed nodes and the mobile node into a two-dimensional distance $\left(d_{1},d_{2},d_{3}\right)$. Then, the trilateral positioning algorithm is used to calculate the robot location coordinates [9].

**Figure 5:**
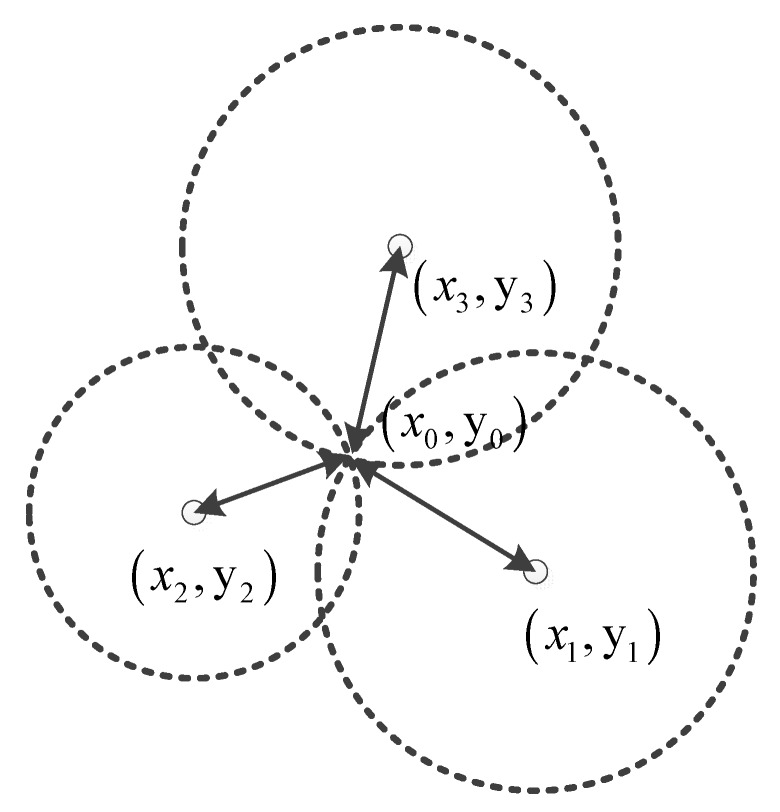
Intersection coordinates.

Figure 5 shows three known points (fixed points) *A*$\left(x_{1},y_{1}\right)$, *B*$\left(x_{2},y_{2}\right)$, and *C*$\left(x_{3},y_{3}\right)$, an unknown point (mobile point) $X\left(x_{0},y_{0}\right)$, and distances $d_{1}$, $d_{2}$, and $d_{3}$ from point *X* to points *A*, *B*, and *C*. Considering three circles with radii of $d_{1}$, $d_{2}$, and $d_{3}$, the coordinates of the unknown point *X* are obtained according to the Pythagorean theorem.

The unknown point is calculated as follows:

(16) $$\begin{array}{l} {\left(x_{1}-x_{0}\right)}^{2}+{\left(y_{1}-y_{0}\right)}^{2}=d_{1}{}^{2}\\ {\left(x_{2}-x_{0}\right)}^{2}+{\left(y_{2}-y_{0}\right)}^{2}=d_{2}{}^{2}\\ {\left(x_{3}-x_{0}\right)}^{2}+{\left(y_{3}-y_{0}\right)}^{2}=d_{3}{}^{2} \end{array}$$

When $(x_{1}-x_{2})(y_{1}-y_{3})-(x_{1}-x_{3})(y_{1}-y_{3})\neq 0$, the solution of be obtained as

(17) $$(x_{0},y_{0})=\left[\begin{array}{l} \frac{C_{1}}{C}(y_{2}-y_{3})+\frac{C_{2}}{C}(y_{3}-y_{1})\\ \frac{C_{1}}{C}(y_{3}-y_{2})+\frac{C_{2}}{C}(y_{1}-y_{3}) \end{array}\right]$$

where $C=2(x_{1}-x_{3})(y_{2}-y_{3})-2(x_{2}-x_{3})(y_{1}-y_{3})$,

$$C_{1}=x_{1}^{2}-x_{3}^{2}+y_{1}^{2}-y_{3}^{2}-d_{1}^{2}+d_{3}^{2}$$

$$C_{2}=x_{2}^{2}-x_{3}^{2}+y_{2}^{2}-y_{3}^{2}-d_{2}^{2}+d_{3}^{2}$$

The accuracy of the UWB positioning system does not decrease with time, as it does not include accumulated errors. However, the system is affected by external interference. Hence, we use a fusion algorithm for the fusion of odometer data and location data with the UWB positioning system to ensure the accuracy of the coordinates in real time.

### 2.4. Fusion of UWB and Odometer Information by Kalman Filtering

As the layout of most restaurants consists of tables and chairs in fixed positions with corridors between them, we set the moving routine of the meal delivery robot according to such a restaurant layout. The robot path width is determined by the width and positioning error of the robot. In this case, the path width is greater than of the robot and includes an error band.

Compared with the odometer location method, the positioning error of the UWB positioning system does not increase with time. There is no accumulated error, but there are some random errors [9]. The fusion algorithm can fuse two positioning methods to exploit the advantages of both. Thus, it can guarantee positioning precision and improve system stability. The proposed fusion algorithm introduces a combined navigation technology based on a Kalman filter, i.e., it combines the odometer location method with ultra-wide band navigation technology through an optimal linear estimation algorithm. In other words, it mutually corrects these complementary technologies. Specifically, it ensures not only timely correction of the odometer location coordinates by addressing the coordinate drift but also effective modification of the ultra-wide band positioning by addressing the random errors and faults. Thus, the dynamic stability and accuracy of the system are improved considerably [14].

Considering that the electronic compass can measure the accuracy of the heading to meet the navigation requirements, using the Kalman filter for the integrated navigation system operation [12] involves only one displacement:

(18) $$\left\{ \begin{array}{l} x_{k}=x_{k-1}+\Delta d_{k,k-1}\cos\theta_{k-1}+\Gamma_{(k,k-1)x}W_{(k-1)x}\\ y_{k}=y_{k-1}+\Delta d_{k,k-1}\sin\theta_{k-1}+\Gamma_{(k,k-1)y}W_{(k-1)y} \end{array}\right.$$

Furthermore,

(19) $$\left\{ \begin{array}{l} z_{x_{k}}=x_{k}+V_{kx}\\ z_{y_{k}}=y_{k}+V_{ky} \end{array}\right.$$

As the intervals between the samples are small, $\theta_{k-1}\approx \theta_{k}$, where *θ* represents the steering angle at time *k* − 1 and *k*.

Here, $W_{(k-1)x},W_{(k-1)y}$ represent the projection process noise sequences, $\Gamma_{(k,k-1)x},\Gamma_{(k,k-1)y}$ are the noise input coefficients, $z_{x_{k}}$, $z_{y_{k}}$ are the robot positioning coordinates calculated by the UWB positioning system, and $V_{kx},V_{ky}$ represent the equivalent noise sequences of the UWB measurement system.

According to the work environment, the process noise sequences and observation noise sequences can be set as random constant-mean Gaussian white noise sequences. Then, we establish a process noise covariance $Q_{k}$ and system noise (measured noise of UWB positioning system) variance $R_{k}$. Throughout the filtering process, the system processes are not related to the noise or noise sequences, i.e., the initial state $x_{0},y_{0}$ of the system is not related to the noise or noise sequences. The initial state of the robot positioning coordinates, $x_{0},y_{0}$, and the initial filtering error value $P_{0}$ are known [19].

Based on the Kalman filter procedure, the odometer navigation system and UWB navigation system undergo the following filter estimation process [14]:

(1) State prediction
  (20) $$\left(\begin{array}{l} {\hat{x}}_{k,k-1}\\ {\hat{y}}_{k,k-1} \end{array}\right)=\left(\begin{array}{l} {\hat{x}}_{k-1}\\ {\hat{y}}_{k-1} \end{array}\right)+\Delta d_{k,k-1}\left(\begin{array}{l} \cos\theta_{k-1}\\ \sin\theta_{k-1} \end{array}\right)$$
(2) State estimation
  (21) $$\left(\begin{array}{l} {\hat{x}}_{k}\\ {\hat{y}}_{k} \end{array}\right)=(\begin{array}{c} \left(1-K_{kx}\right)\\ 0 \end{array}\begin{array}{c} 0\\ \left(1-K_{ky}\right) \end{array})\left(\begin{array}{l} {\hat{x}}_{k,k-1}\\ {\hat{y}}_{k,k-1} \end{array}\right)+\left(\begin{array}{l} K_{kx}z_{x_{k}}\\ K_{ky}z_{y_{k}} \end{array}\right)$$
(3) Filter gain
  (22) (KkxKky)=(P(k,k−1)x[P(k,k−1)x+Rkx]P(k,k−1)y[P(k,k−1)y+Rky])
(4) Step error
  (23) $$\left(\begin{array}{l} P_{(k,k-1)x}\\ P_{(k,k-1)y} \end{array}\right)=\left(\begin{array}{l} P_{(k-1)x}\\ P_{(k-1)y} \end{array}\right)+\left(\begin{array}{l} \Gamma^{2}{}_{(k,k-1)x}Q_{(k-1)x}\\ \Gamma^{2}{}_{(k,k-1)y}Q_{(k-1)y} \end{array}\right)$$
(5) Estimation based on error
  (24) $$\left(\begin{array}{l} P_{kx}\\ P_{ky} \end{array}\right)=(\begin{array}{c} {\left[1-K_{kx}\right]}^{2}\\ 0 \end{array}\begin{array}{c} 0\\ {\left[1-K_{ky}\right]}^{2} \end{array})\left(\begin{array}{l} P_{(k,k-1)x}\\ P_{(k,k-1)y} \end{array}\right)+\left(\begin{array}{l} K_{kx}^{2}R_{kx}\\ K_{ky}^{2}R_{ky} \end{array}\right)$$

During this robot localization via fusion, the above-mentioned formula can be obtained using the robot’s current position coordinates by selecting the noise variances $Q_{k}$ and $R_{k}$.

### 2.5. Extended Kalman Filter Fusion

Owing to the presence of external interference and noise, a measurement error exists in the sensor itself as well as in the model. The traditional Kalman filter fusion algorithm introduced above cannot eliminate these errors in practical engineering applications [26]. Hence, the result will not be the optimal solution. At the same time, only a linear system can use the traditional Kalman filter fusion algorithm, and the noise must be Gaussian white noise. However, this is not straightforward in practical engineering applications. Therefore, we propose an optimized Kalman filter fusion algorithm.

Among the various non-linear filtering techniques, the EKF fusion algorithm is the simplest algorithm that locally linearizes the Kalman filter fusion algorithm and is suitable for weakly nonlinear [27,28], non-Gaussian white noise environments. The EKF fusion algorithm performs a first-order Taylor expansion of the nonlinear function of the system and obtains a linearized system formula to complete the filtering estimation of the target [29].

Considering discrete-time nonlinear systems, the state and measurement formula are

(25) $$x_{k+1}=f_{k}(x_{k},\omega_{k})=\phi x_{k}+Q$$

(26) $$z_{k}=h_{k}(x_{k},v_{k})=h(x_{k})+v$$

Assuming that the state formula is a linear function and the measurement formula is a nonlinear function, and that the state estimation value ${\hat{x}}_{k/k-1}$ and estimated error covariance matrix $P_{k-1/k-1}$ at time k−1 have been obtained, the steps of the first-order EKF filtering fusion algorithm are as follows.

According to the known state equation, expand the target state to obtain ${\hat{x}}_{k/k-1}$.

(1) Apply Taylor expansion to the nonlinear measurement formula h(x) at ${\hat{x}}_{k/k-1}$ and keep the first item of the formula. Thus, we can obtain

(27) $$h(x_{k/k})\approx h({\hat{x}}_{k/k-1})+\frac{\partial h}{\partial h^{T}}{|}_{x={\hat{x}}_{k/k-1}}(x-{\hat{x}}_{k/k-1})$$

(28) $$h(x_{k/k})=h({\hat{x}}_{k/k-1})+H({\hat{x}}_{k/k-1})(x-{\hat{x}}_{k/k-1})$$

where $H({\hat{x}}_{k/k-1})=\frac{\partial h}{\partial x^{T}}{|}_{x={\hat{x}}_{k/k-1}}$ is the Jacobian matrix of h(x).

Equation (27) is substituted into Equation (28) to get the approximate linearization model:

(29) $$z=H({\hat{x}}_{k/k-1})x+h({\hat{x}}_{k/k-1})-H({\hat{x}}_{k/k-1}){\hat{x}}_{k/k-1}+v$$

(2) The state filtering update of the gain matrix and error covariance matrix are

(30) $$K=P_{k/k-1}H^{T}{\left(HP_{k/k-1}H^{T}+R\right)}^{-1}$$

(31) $${\hat{x}}_{k/k}={\hat{x}}_{k/k-1}+K[z-z({\hat{x}}_{k/k-1})]$$

(32) $$P_{k/k}=(I-KH)P_{k/k-1}{\left(I-KH\right)}^{T}+KRK^{T}$$

As the measurement matrix is replaced by the Jacobian matrix H, the gain K calculated from Equation (30) is not the optimal gain. Therefore, the traditional calculation formula for the matrix *P* cannot be used to update the covariance matrix, and Equation (32) must be used to ensure positive-definiteness and symmetry of *P*. This is the only way to prevent the filter from diverging, and it is also the only way to guarantee convergence.

The accuracy of the EKF fusion algorithm using first-order Taylor expansion depends on the target state dynamic model and the previous state estimation. If $x-{\hat{x}}_{k/k-1}$ is sufficiently small, the output of the system is the optimal solution.

The UWB positioning system used in this experiment measures the distance between the reference node and the moving node using the time difference of arrival (TDOA) algorithm and two-way time-of-flight measuring principle. Assuming that the reference node A of the positioning system sends a distance measurement command to the reference node B at time $\tau_{txa}$, the reference node B receives the instruction from the reference node A and returns a corresponding response command at time $\tau_{rxb}$. The reference node A receives the response command from the reference node B at the $\tau_{rxa}$ and calculates the time difference of the UWB signal from time $\tau_{txa}$ to time $\tau_{rxa}$. The product of this time difference and the UWB signal speed $v_{UWB}$ is the distance between the reference node A and the reference node B, $d_{AB}$. Hence, the formula for obtaining the distance is as follows:

(33) $$d_{AB}=v_{UWB}\times [(\tau_{rxa}-\tau_{txa})-(\tau_{rxb}-\tau_{txb})]/2$$

In the line-of-sight (LOS) environment, ${\hat{d}}_{0,i}$ is assumed as the measured distance between reference point $P_{0}$ and reference point $P_{i}$. Then, the true value of the distance is

(34) $${\hat{d}}_{0,i}=d_{0,i}+v_{0,i}(d_{0,i})$$

where $v_{0,i}(d_{0,i})$ is an absolute error function. This function is related to the actual distance. According to the actual application of the delivery robot, this study assumes that this function is a linear function.

(35) $$v_{0,i}(d)=a_{0,i}d+b_{0,i}$$

(36) $$d_{0,i}=({\hat{d}}_{0,i}-b_{0,i})/(1+a_{0,i})$$

**Figure 6:**
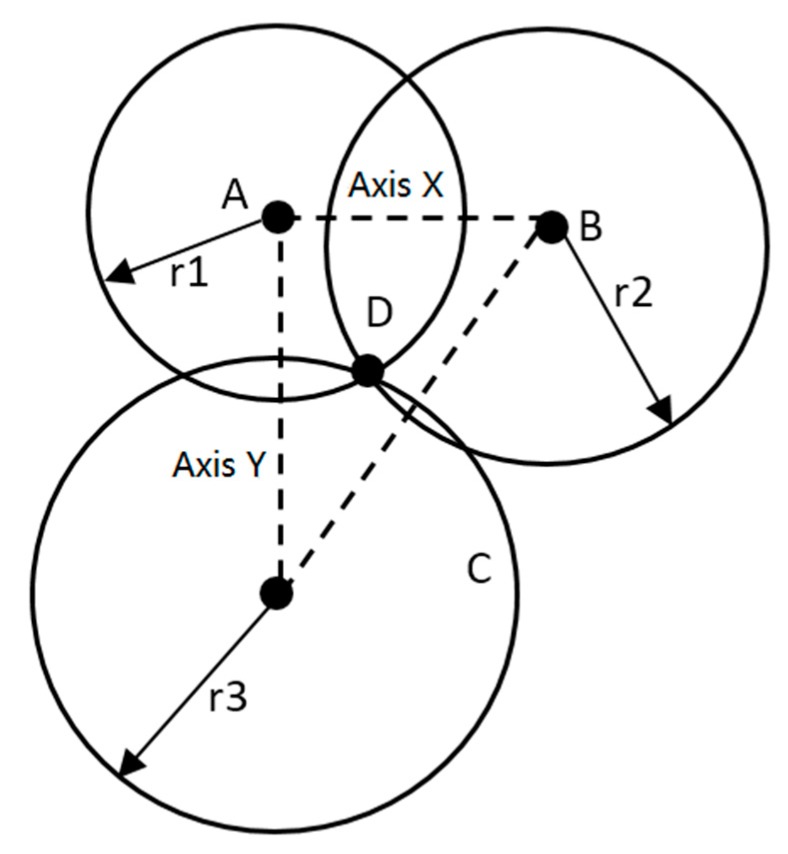
Schematic of time difference of arrival (TDOA) positioning algorithm.

In practice, there are many obstacles in the working environment of the delivery robot. The environment must be a non-line-of-sight (NLOS) environment. NLOS errors will cause interference, diffraction, and other phenomena that affect the UWB signal. Hence, there are errors in the time difference between reference nodes receiving the signal. Therefore, we assume that the indoor environment of the experimental platform does not change and that the obstacles in the environment are all of the same material so that the NLOS error can be regarded as a fixed constant. Then, the distance formula is rewritten as

(37) $$d_{0,i}=\frac{{\hat{d}}_{0,i}-b_{0,i}}{1+a_{0,i}}-J\cdot v_{NLOS},\left\{ \begin{array}{l} SNR\geq T_{SNR},J=0\\ SNR\leq T_{SNR},J=1 \end{array}\right.$$

where $v_{NLOS}$ is the *NLOS* error constant in the indoor environment. The signal-to-noise ratio (SNR) is the ratio between the signal received by the mobile nodes and the system noise, while $T_{SNR}$ is the ranging condition determination threshold. The UWB positioning system used in this experiment employs the TDOA positioning algorithm. To ensure accurate calculation of the arrival time difference, the algorithm must ensure that the reference nodes of the UWB positioning system are at the same height. As shown in Figure 6, reference node A is the origin of the coordinate system, the line connecting reference node B and reference node C is the *X*-axis, and the line connecting reference node A and reference node C is the *Y*-axis. The *Z*-axis direction is perpendicular to the horizontal plane formed by the reference nodes A, B, and C.

From Figure 6, we can see that the position of the intersection of the three circles is the position of the mobile node D. Assuming the coordinates as (x,y,z), the coordinates of the reference nodes A, B, and C are $\left(x_{i},y_{i},z_{i}\right)$, where i=1,2,3. According to the Euclidean distance formula, the distance between the mobile node and the reference node is calculated as

(38) $$r_{i}=\sqrt{{\left(x_{i}-x\right)}^{2}+{\left(y_{i}-y\right)}^{2}+{\left(z_{i}-z\right)}^{2}}$$

According to Equation (33), the coordinates of the moving node and measured distance are not linearly related. Therefore, the measurement formula is a non-linear equation. Therefore, the EKF fusion algorithm is used to improve positioning accuracy.

Assume that the state formula of the system’s reference node A is

(39) $$X_{k}=f_{k-1}(X_{k-1})+W_{k-1},W_{k}\sim N(0,Q_{k})$$

The nonlinear measurement formula is

(40) $$Z_{k}=h_{k}(X_{k})+V_{k},V_{k}\sim N(0,R_{k})$$

where $X_{k}$ is the velocity vector of the moving node relative to the coordinate system. This vector orthogonally decomposes the corresponding velocity and corresponding coordinates of the X,Y,Z axes.

(41) $$X_{k}={\left[x(k),y(k),z(k),v_{x}(k),v_{y}(k),v_{z}(k)\right]}^{T}$$

(42) $$v_{i}(k)=\frac{\left(i(k)-i(k-1)\right)}{T},i=x,y,z$$

In Equation (39), $Q_{k}$ is the covariance matrix of the prediction noise vector $W_{k}$, and $Z_{k}$ is the measurement distance vector between the reference nodes and the moving node, which is concretely expressed as

(43) $$Z_{k}={\left(r_{1}(k),r_{2}(k),r_{3}(k)\right)}^{T}$$

where $r_{k}$ is the covariance matrix of the noise vector $v_{k}$ and T is the sampling period of the system. Then, the equations of the EKF fusion algorithm are as follows:

(44) $${\hat{X}}_{\bar{k}}=f_{k-1}({\hat{X}}_{k-1})$$

(45) $$P_{\bar{k}}=\varphi_{k-1}P_{k-1}\varphi_{k-1}^{T}+Q_{k}$$

(46) $$K_{k}=P_{\bar{k}}H_{k}^{T}{\left(H_{k}P_{\bar{k}}H_{k}^{T}+R_{k}\right)}^{-1}$$

(47) $${\hat{X}}_{k}={\hat{X}}_{k}+{\hat{K}}_{k}({\hat{Z}}_{k}-h_{k}({\hat{X}}_{\bar{k}}))$$

(48) $$P_{k}=(I-K_{k}H_{k})P_{\bar{k}}$$

where $K_{k}$ is the gain calculation matrix of the EKF fusion algorithm, $P_{k}$ is the system error’s covariance matrix, and $\varphi_{k-1}\approx \frac{\partial f_{k}}{\partial x}|x={\hat{X}}_{\bar{k}-1}$ is a system state transition matrix. Further, $f_{k-1}(X_{k-1})=(\begin{array}{l} I_{3}\\ 0 \end{array}\begin{array}{l} TI_{3}\\ I_{3} \end{array})$, where $I_{3}$ is a third-order identity matrix and $H_{k}$ is the Jacobian matrix of $h(k)(X_{k})$ at time k.

### 2.6. Meal Delivery Robot Trajectory Control

To obtain accurate and stable location coordinates, we developed a special trajectory according to the positioning algorithm.

Considering that the restaurant corridors are often extremely narrow and that the obstacles are usually pedestrians, the robot will stop when it encounters the obstacles and prompt the pedestrians to move. In this case, the infrared and ultrasonic obstacle detection module detects obstacles in front and on the sides.

**Figure 7:**
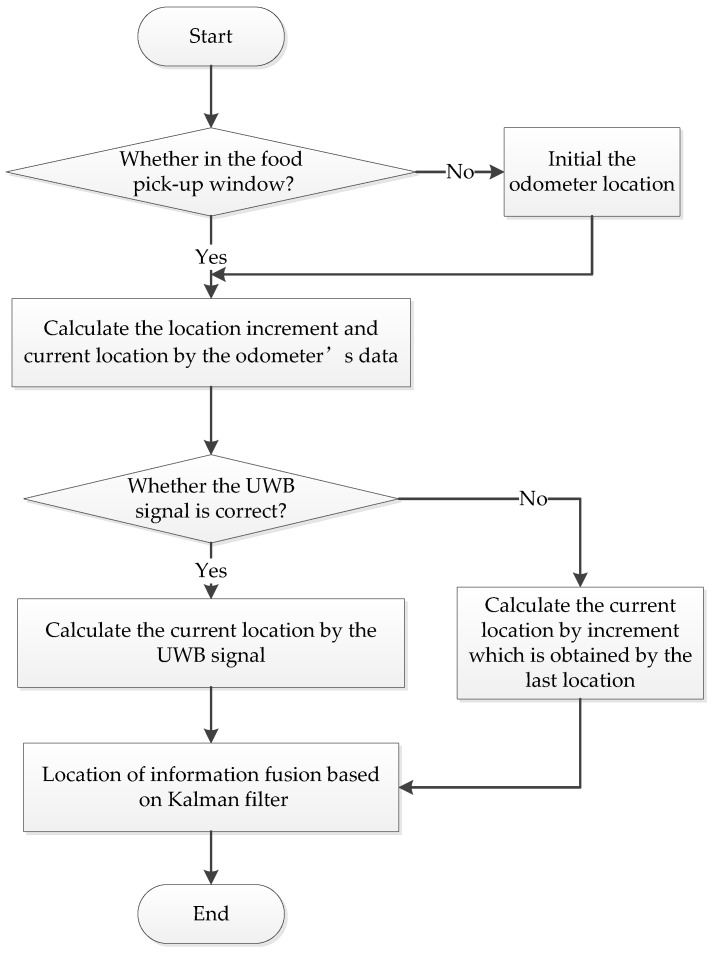
Delivery robot control flowchart.

**Figure 8:**
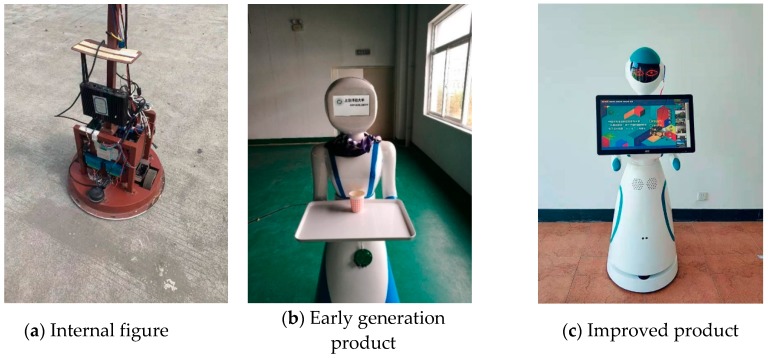
Food Delivery Robot.

The path of the meal delivery robot is set as a fixed path by the embedded software according to the location selected. The robot control processes are shown in Figure 7 and the real product is shown in Figure 8. In Figure 8a is the internal structure drawing of robot, Figure 8b is the original food delivery robot, and Figure 8c is the modified product drawing.

The motion control schedule includes the following steps:

(1) According to the restaurant layout, the tables’ location coordinates and meal delivery robot trajectory coordinates are confirmed.
(2) Initially, the odometer heading and attitude sensor data are cleared to ensure that no cumulative error exists.
(3) The EKF fusion algorithm is used to get accurate real-time coordinates via the UWB GPS coordinates and odometer.
(4) Considering the actual error caused by a “single cumulative error”, the actual driving process adjusts the attitude heading sensor readings in real time to determine the direction of the robot if the error is less than the allowable error [17].

During the implementation of the fusion algorithm, the system needs to determine whether the UWB positioning system is operating properly by judging the robot movement velocity and displacement. For example, if the robot speed is greater than zero, the displacement of the control cycle is changed. Meanwhile, if the UWB positioning system moves to a certain distance from each reference node, the information on the incremental position and the last position is used for the current real-time location coordinates. If the UWB signal is correct, the odometer positioning result is fused with the UWB localization result using the EKF algorithm to get the current position coordinates in real time [19].

During motion, the system error and random errors may cause a shift in the trajectory of the robot. Hence, adjustment is required. Within the predetermined track range, the running attitude is maintained. Outside the predetermined track range, the running attitude is adjusted. The adjustment method is as follows. If the robot moves to the left (right) beyond the predetermined trajectory, the left (right) wheel speed will be increased. Using the fuzzy logic control strategy, the system achieves real-time online fuzzy control in the case of the speed difference between the left and right wheels [18].

## 3. Experiment and Result Analysis

### 3.1. Experimental System

We design a comparative experiment as follows. We use the ROS platform as a control platform to control the delivery robot motion along the reference trajectory, and we obtain the coordinates of the UWB positioning system as well as those of the improved odometer positioning method. When the improved odometer positioning method is used, the heading angle of the robot must be calculated and measured by the IMU. After obtaining two sets of coordinates, the EKF fusion algorithm is used to fuse the data in order to verify the optimization of accuracy and stability. The diameter of the drive wheel of the delivery robot is 12 cm, and the distance between the left and right wheels is 34 cm.

**Figure 9:**
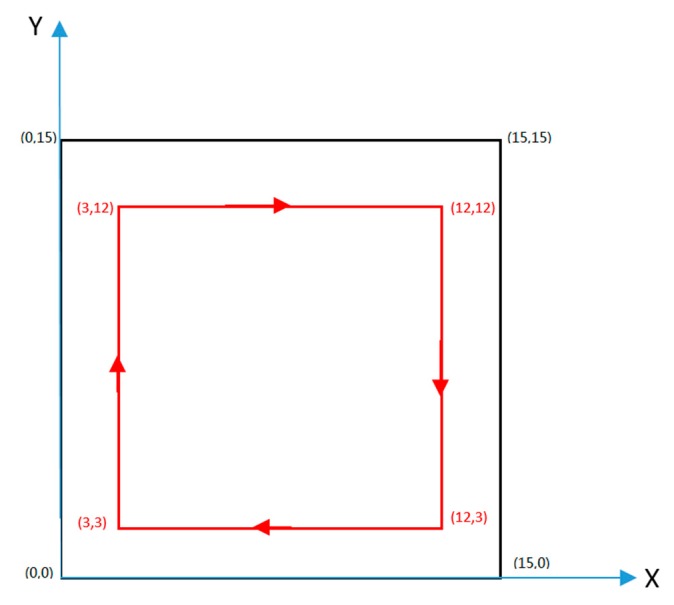
Delivery robot operation site diagram.

Figure 9 shows a schematic diagram of the operation site. The experimental environment is a square room with a side length of 15 m. The environment has no special requirements, so there is no detailed description. Although the robot has the infrared and ultrasonic obstacle detection module, the obstacle avoidance function is not included in the study. In addition, the obstacles are usually pedestrians. There is no barrier in the experiment room except for some tables and chairs. The operators are in the room but not on the robot’s trajectory and they may affect UWB signal reception. The environment was similar to that of a restaurant. The reference nodes of the UWB positioning system are respectively placed at the four vertices of the square region, and the *x*-*y* two-dimensional coordinate system is established with reference node 1 as the origin. The remote control delivery robot starts from the point (3,3) and finally returns to this starting point via the points (3,12), (12,12), and (12,3). The walking trajectory is the edge of a square whose side is 9 m long.

During the experiment, the data sampling frequencies of the UWB positioning system, IMU, and odometer are all 10 Hz.

### 3.2. UWB Positioning System Position

**Figure 10:**
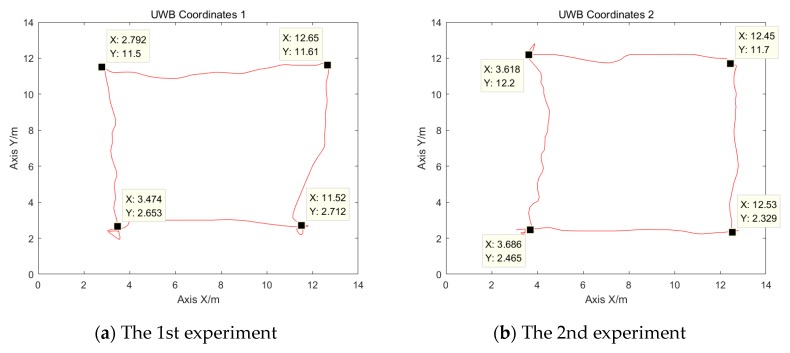

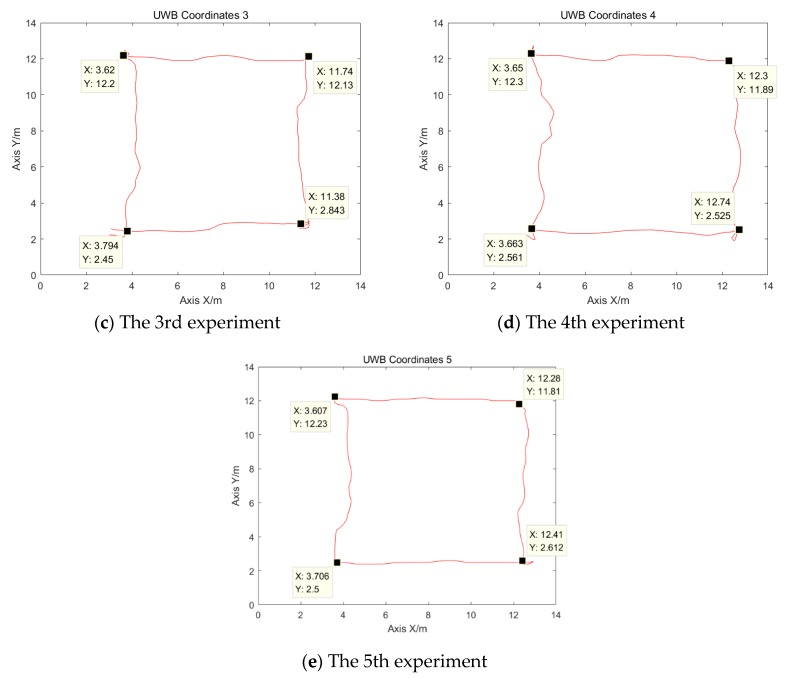
The tracks of the delivery robot positioned by the ultra-wide band (UWB) positioning system.

The mobile node of the UWB positioning system is installed on the head of the robot and the reference nodes are deployed at the four top corners of a square whose side is 15 m long. The installation height of the reference nodes is 2.25 m. The computer software reads the position coordinates of the UWB positioning system using UDP. The control signal is sent to the main control board through the ROS platform in order to control the motor so that the delivery robot runs along the red track in Figure 8. Specifically, the overall verification experiment was conducted by five groups of experiments in two different situations. Situation I is comprised of three groups of experiments and the number of people within the scope of the experimental area is less than four. While situation II is made up of two groups of experiments and there are more than 10 people within the experimental area. In addition, the other obstacles such as tables and chairs are identical in both situations. The track of the delivery robot positioned by the UWB positioning system is shown in Figure 10.

Figure 10a–e show the 5 times track of the delivery robot positioned by the ultra-wide band (UWB) positioning system. From Figure 10 it can be seen the coordinate data indicates that the accuracy and stability of the positioning coordinates obtained by the UWB positioning system are poor. The main reasons are as follows:

(1) Although the TDOA algorithm is used in the UWB positioning system, it is difficult to achieve full synchronization in the initial state owing to the influence of the hardware circuits (mainly, crystal oscillators) and temperature. With time, clock drift will be generated, and the original synchronous clock system becomes unsynchronized. Although the system corrects this error, it still affects the final positioning accuracy.
(2) In the UWB signal transmission process, it is difficult to ensure that the environment is completely LOS. The signal will be reflected and refracted owing to obstacles and other factors, and it will become NLOS. This will lead to a reduction in the final positioning accuracy.
(3) Because the system in an indoor environment and the moving node is close to the motor power supply, the noise level of the entire system is increased, which reduces the positioning accuracy. At the same time, the randomness of the noise itself may lead to the appearance of some anomalies.

### 3.3. Coordinate Calculation for Improved Odometer Positioning Method

When the improved odometer positioning method calculates the coordinates, it needs to use the heading angle calculated by the traditional odometer positioning method and the heading angle measured by the IMU as the input. Therefore, the heading angle is first calculated to obtain two sets of heading angles. Then, the coordinates are calculated.

(1) IMU heading angle measurement

**Figure 11:**
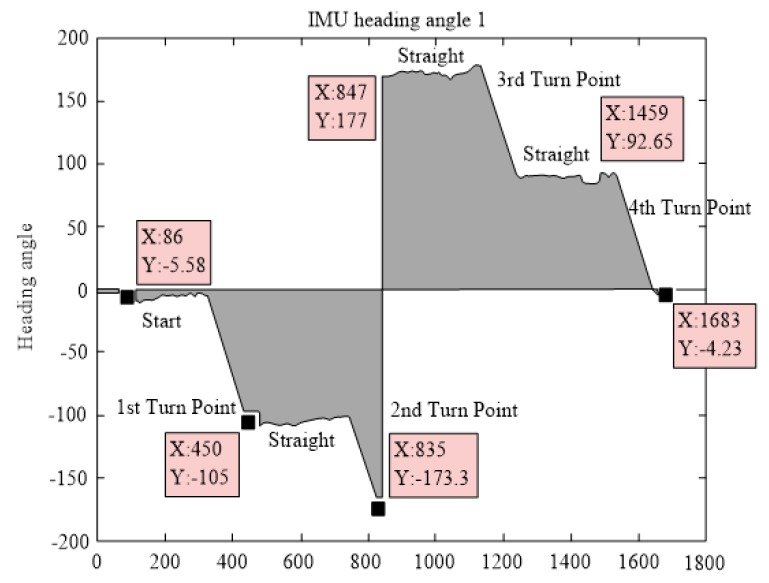
Heading angle changes measured by one of five inertial measurement units (IMUs).

The heading angle was measured by the IMU and five sets of experimental data were obtained. One set of heading angle changes as shown in Figure 11.

In Figure 11, the first stage is the start-up operation stage. At this time, the delivery robot started and moved to the first 90° turning point. In this process, the theoretical heading angle measured by the IMU is 0°.

**Figure 12:**
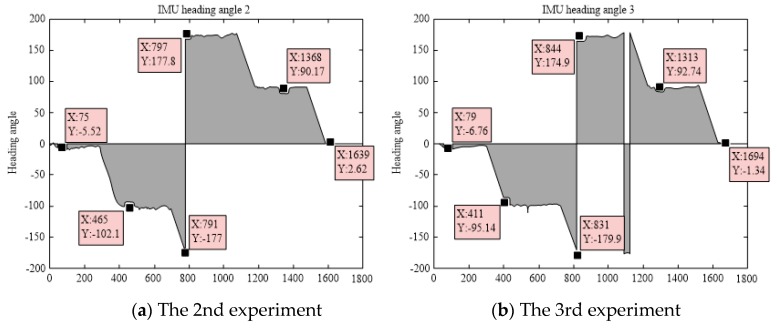

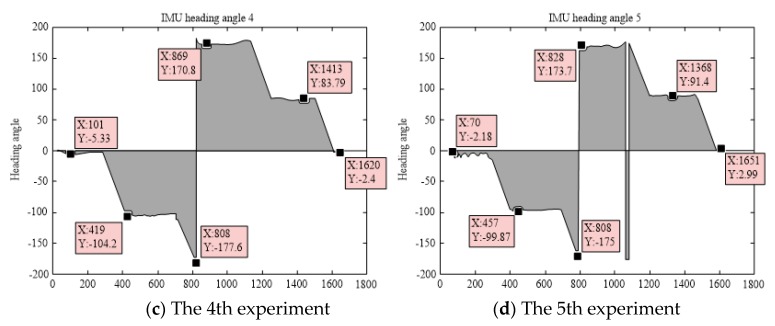
Heading angle changes measured by other four IMUs.

In the second stage, the delivery robot rotated through 90°, and the theoretical heading angle after turning was −90°. Then, the robot moved in a straight direction again, reached the second turning point, and rotated through 90°. At this time, the theoretical heading angle should be 180°. The measurement data of the IMU has errors, and the theoretical range of its measurement values is (−180°, 180°]. Hence, measurement data similar to the step signal will be generated at a specific time. The delivery robot will then return to the starting point via the remaining turning points and the straight path. Figure 12 shows the rest 4 sets of heading angles measured by the IMU.

The main error of the IMU comes from the following sources.

(i) Vibration caused by the motion of the delivery robot: When the accelerometer works with the vibration interference, its measurement error will become larger.
(ii) Electromagnetic and metal interferences during the operation of the robot: Although the IMU has a filtering algorithm to filter the electromagnetic interference, the distance between the servo motor and the sensor is fairly short, which affects the measurement precision of the IMU. In addition, the IMU has been calibrated to be insensitive to the metal material of the delivery robot, errors may still be induced during the motion of the robot.
(iii) The gyroscope in the IMU will have zero drift, which means that even with a heading angle of 0°, the unit will have an output. At the same time, the measurement unit’s data will be affected by the temperature.

(2) Traditional odometer positioning method heading angle measurement

When the traditional odometer positioning method calculates the heading angle, the time interval between the initial time t0 and t1 is set as the sampling period, which is 100 ms. The odometer data QCL and QCR of the left and right wheels are sampled every 100 ms.

**Figure 13:**
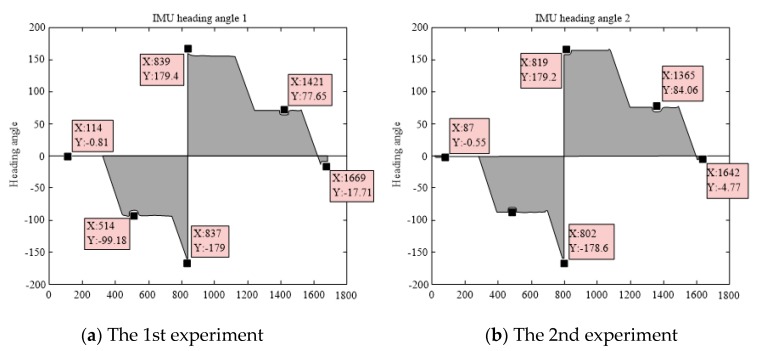

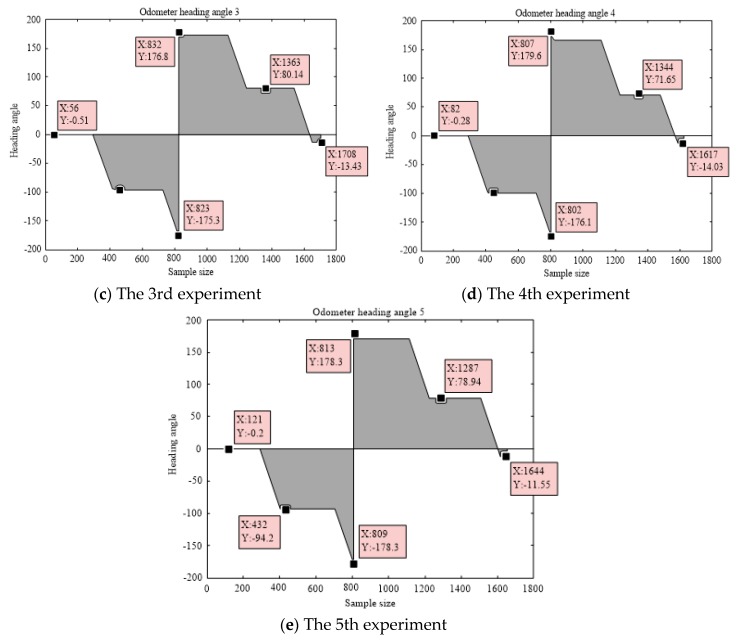
Schematic diagram of the change in the heading angle calculated by the improved odometer positioning method.

The traditional odometer positioning method was used in five groups of experiments to calculate the heading angle. Figure 13 shows the calculated heading angle data of 5 experiments.

As can be seen from Figure 13, the heading angle calculated by the odometer data is highly accurate in the beginning, but the error increases with the distance owing to the accumulated error.

The final cumulative error comes from two sources. The first one is the floating-point error that is generated when calculating the heading angle from the formula. Considering the computing power of the main control chip, only two decimal places are reserved for each calculation of the heading angle. The heading angle is also obtained by accumulating the heading angle variations Δθ during the sampling period Δt, which will lead to greater errors. The second source is the accuracy error of the odometer itself and the measurement error during the experiment.

### 3.4. Positioning Coordinate Calculation

**Figure 14:**
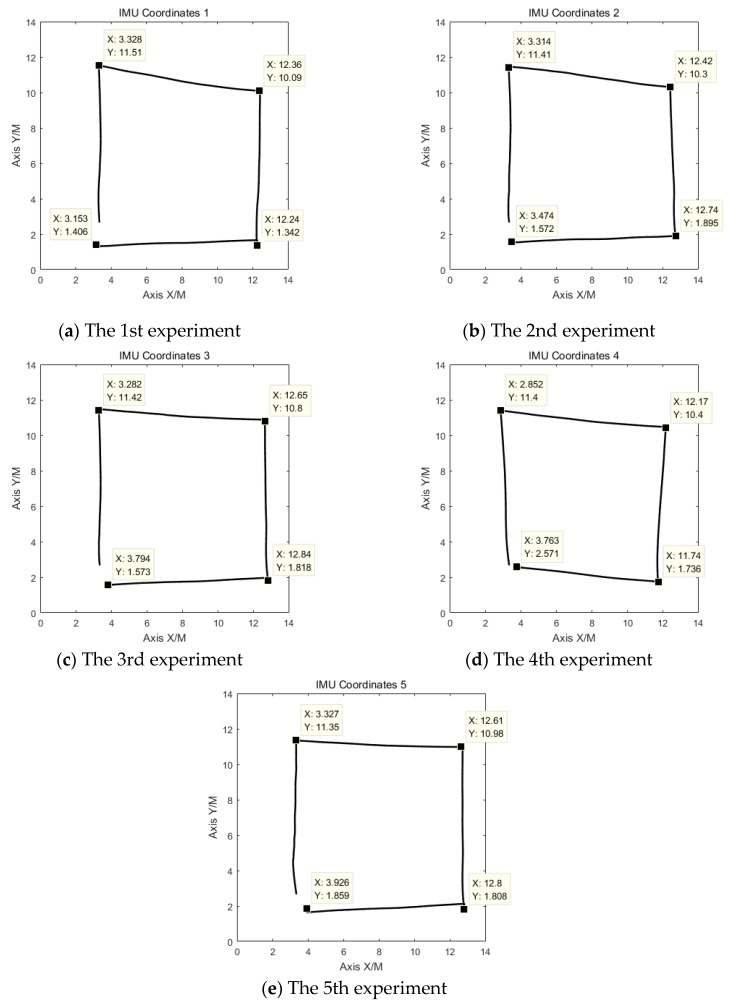
Track of the delivery robot positioned by the improved odometer positioning method.

The heading angle measured by the IMU and the heading angle calculated by the odometer data in the sampling period were used as the input parameters of the improved odometer positioning method to calculate the coordinates. The experiments were performed five times. Figure 14 shows the coordinate data obtained by the improved odometer positioning method.

From Figure 14, the heading angles measured by IMU and by the odometer data are used to calculate the coordinates of the robot, the connection part of the coordinate image is not a straight line. This is because the heading angle measured by the IMU changes under the influence of the error. Considering that the IMU is sensitive to magnetic fields and metals, the data will still fluctuate when these two interference sources are suddenly encountered. Possible sources of interference are metal shelves, elevators, etc. The stability of the IMU is poor, and it is easily affected by environmental factors. The heading angle measured by the IMU will affect the final positioning coordinate data of the improved odometer positioning method.

The improved odometer positioning method combines the IMU, which can accurately reflect the heading angle, and the odometer, which calculates the heading angle with a small error. It can obtain better coordinates in the case of long-duration and long-distance operation. However, the IMU data are susceptible to interference and large fluctuations. Hence, the independent use of the improved odometer positioning method is not reliable.

### 3.5. EKF Fusion Algorithm Coordinate Fusion Experiment

**Figure 15:**
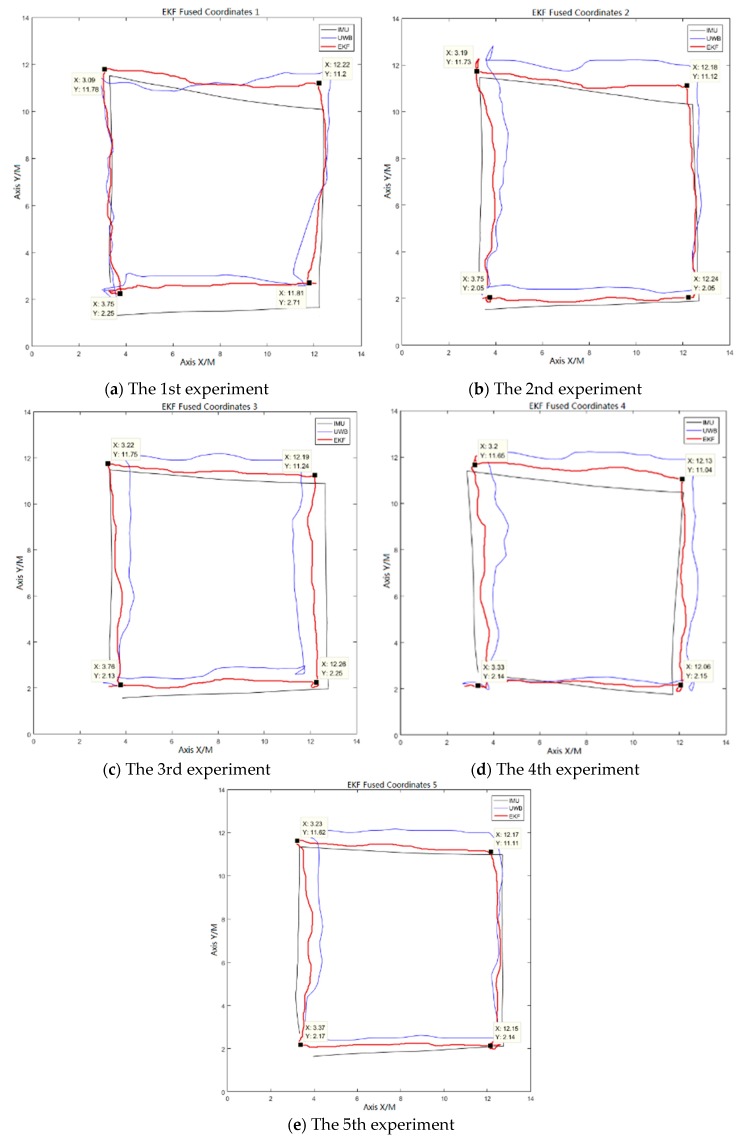
Extended Kalman filter (EKF) fused coordinates and original coordinates comparison.

In this experiment, the positioning results of the UWB positioning system are fused with those of the improved odometer positioning method to obtain the positioning results with better accuracy and stability, closer to the actual situation. The positioning results of the improved odometer positioning method are used as the observations of the EKF algorithm, and the positioning results of the UWB positioning system are used as the state variables of the EKF algorithm. We set the initial parameters and status of the system as follows: system noise Q = 0.4, the variance of the measurement noise of the UWB positioning system R = 0.35. The sampling frequency of both methods is 10 Hz. After the EKF fusion algorithm is executed, the fusion coordinates are obtained. Figure 15 compares the EKF fusion coordinates with the original coordinates.

From Figure 15, it can be clearly seen that the accuracy and stability of the fusion positioning results have improved. According to the positioning error calculation formula, the distance of the actual positioning coordinates from the ideal coordinates can be calculated. This distance value reflects the positioning accuracy. The error calculation formula is as follows:

(49) $$E_{s}=\sqrt{{\left(X_{i}-X\right)}^{2}+{\left(Y_{i}-Y\right)}^{2}}$$

where $E_{s}$ is the distance of the measured coordinates or fused coordinates from the ideal coordinates, X and Y are the horizontal and vertical values of the ideal coordinates, and $X_{i}$ and $Y_{i}$ are the horizontal and vertical values of the measured or fused coordinates.

**Figure 16:**
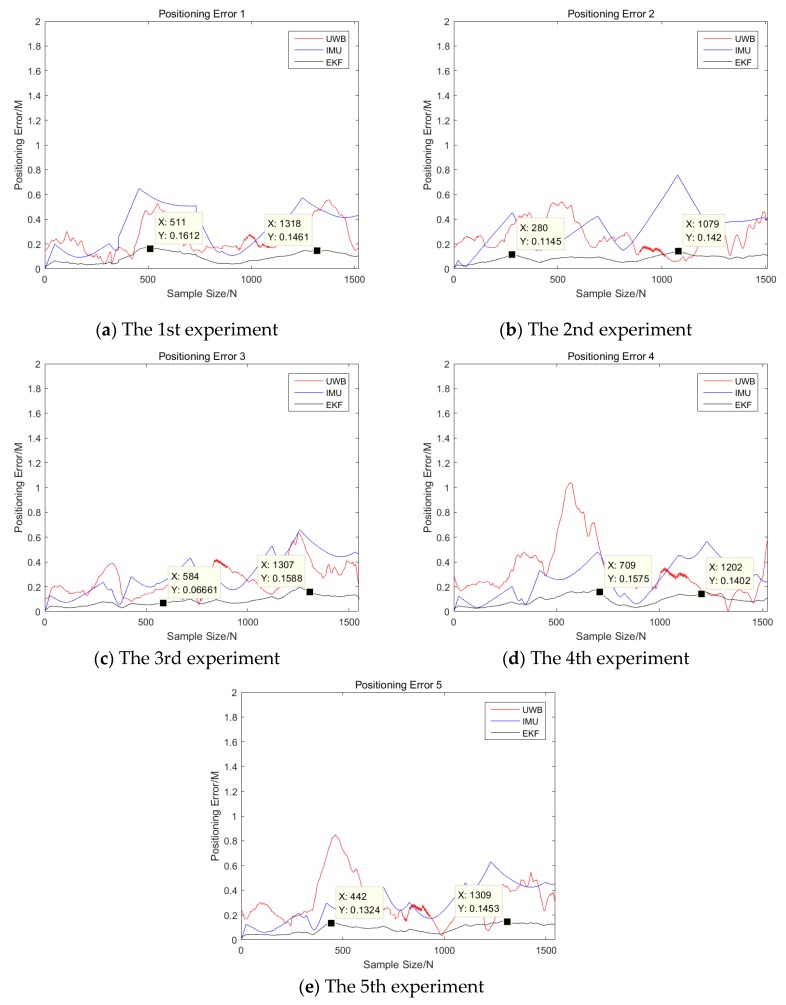
Positioning error comparison.

Figure 16 shows the 5 experiments’ error comparison of the coordinates of the UWB positioning system, the improved odometer positioning method, and the EKF fusion algorithm. To be more intuitively, the statistical results of the maximum position errors of the three positioning methods are listed in Table 1.

From Table 1, it can be seen that the position error of the EKF fusion algorithm is far less than using the IMU or UWB. In addition, the number of indoor people has little effect on the accuracy of the UWB and IMU integrated system. On the other hand, the accuracy of UWB alone is sensitive to the number of people in the room. As the number of people in the room increases, the accuracy of using UWB alone will be decreased. This also verified the effect of the human body on the UWB signal reception. From the table, it can also be seen that the number of people has little effect on the IMU, which is consistent with the characteristics of the IMU.

The following conclusions can be drawn from the coordinate comparison and error comparison. The UWB positioning system has poor positioning stability and low positioning accuracy. Especially when the number of indoor people or things increases, the accuracy is further reduced. When it is used independently, it is difficult to provide reliable coordinate data for the delivery robot. The improved odometer positioning method has better positioning results, and the error of the start-up phase of the delivery robot is also smaller. However, with time, the cumulative error increases. Thus, the final positioning accuracy decreases. The stability and accuracy of the positioning coordinates after fusion by the EKF fusion algorithm are improved considerably compared to the independent use of the first two methods. In particular, the positioning error of the fused coordinates is less than 15 cm.

This experiment verified the effect of the EKF fusion algorithm on the positioning results. However, considering the poor positioning stability of the UWB positioning system and the accumulated error of the improved odometer positioning method, the final error of the fused positioning coordinates is still high. Therefore, although the fused positioning results have been improved compared to the use of a single positioning method, there is scope for further optimization.

## 4. Conclusions

The growing popularity of robots in the service industry can be attributed to their low labor cost, high efficiency, and attractiveness to customers. Nevertheless, the development and improvement of delivery robots are imperative. In this study, according to the coordinate comparison diagram and error comparison diagram, it can be concluded that the positioning result of the UWB positioning system is of poor stability and low positioning accuracy. When UWB is used independently, it is difficult to provide reliable coordinate data for the food delivery robot. The positioning results of the improved track deduction algorithm are stable and the error of the food robot is small at the beginning of operation. However, with the increase of time, the cumulative error gradually increases and the final positioning accuracy is low. The fusion method of EKF is used to improve the stability and accuracy of the fusion coordinates, and the positioning error is less than 15 cm which can be accepted in a restaurant. Based on this method, the delivery robot has high positioning accuracy, low installation and maintenance costs, wide availability, and low impact on the restaurant layout. Moreover, it is simple and flexible in terms of changing the travel route. Future work will focus on enabling enable multiple delivery robots on site.

## Figures and Tables

**Table 1:** The maximum error statistical results of the three positioning methods.

Positioning Methods Experimental Cases		IMU	UWB	EKF
Situation I	1(a)	63.1 cm	59.6 cm	16.2 cm
	2(b)	79.9 cm	58.4 cm	14.2 cm
	3(c)	63.3 cm	62.6 cm	14.7 cm
Situation II	4(d)	59.4 cm	103.5 cm	15.5 cm
	5(e)	61.3 cm	82.3 cm	15.0 cm

## References

[1] Zhou L. (2015). Application of restaurant service robot. Wind Sci. Technol..

[2] Wakita Y., Tanaka H., Matsumoto Y. Projection Function and Hand Pointerfor User Interface of Daily Service Robot. Proceedings of the 2017 IEEE International Conference on Robotics and Biomimetics (ROBIO).

[3] Kantharak K., Somboonchai C., Tuan N.T., Thinh N.T. Design and development of service robot based human-robot interaction (HRI). Proceedings of the International Conference on System Science and Engineering.

[4] Hernandez-Mendez S., Maldonado-Mendez C., Marin-Hernandez A., Rios-Figueroa H.V. Detecting falling people by autonomous service robots: A ROS module integration approach. Proceedings of the International Conference on Electronics, Communications and Computers.

[5] Shin H., Chon Y., Kim Y., Cha H. (2015). A participatory service platform for indoor location-based services. IEEE Pervasive Comput..

[6] Xin J., Jiao X.L., Yang Y., Liu D. Visual navigation for mobile robot with Kinect camera in dynamic environment. Proceedings of the Chinese Control Conference.

[7] Gulalkari A.V., Sheng D., Pratama P.S., Kim H.K., Byun G.S., Kim S.B. Kinect camera sensor-based object tracking and following of four wheel independent steering automatic guided vehicle using Kalman filter. Proceedings of the International Conference on Control, Automation and Systems.

[8] Haiyu L., You L., Naser E.S. (2015). Pdr/ins/wifi integration based on handheld devices for indoor pedestrian navigation. Micromachines.

[9] Woods J.O., Christian J.A. (2016). Lidar-based relative navigation with respect to non-cooperative objects. Acta Astronaut..

[10] Xujian H., Hao W. WIFI indoor positioning algorithm based on improved Kalman filtering. Proceedings of the International Conference on Intelligent Transportation, Big Data & Smart City (ICITBS).

[11] Zhang W., Hua X., Yu K., Qiu W., Zhang S. Domain clustering based WiFi indoor positioning algorithm. Proceedings of the International Conference on Indoor Positioning and Indoor Navigation (IPIN).

[12] Yu K., Wen K., Li Y., Zhang S., Zhang K. (2018). A Novel NLOS Mitigation Algorithm for UWB Localization in Harsh Indoor Environments. IEEE Trans. Veh. Technol..

[13] Rahman M.A., Reaz M.B.I., Husain H., Ali M.A.B.M., Marufuzzaman M. (2013). Performance analysis of Bluetooth Zigbee and Wi-Fi protocols in 2.4 GHz multi-standard Zero-IF receiver. Przeglad Elektrotechniczny.

[14] Ni W., Wang Z.X. (2006). Indoor location algorithm based on the measurement of the received signal strength. Front. Electr. Electron. Eng. China.

[15] Dardari D., Conti A., Ferner U., Giorgetti A., Win M.Z. (2009). Ranging with ultrawide bandwidth signals in multipath environments. Proc. IEEE.

[16] Guan L., Cong X., Sun Y., Gao Y., Iqbal U., Noureldin A. (2017). Enhanced MEMS SINS Aided Pipeline Surveying System by Pipeline Junction Detection in Small Diameter Pipeline. IFAC-PapersOnLine.

[17] Krishnan S., Sharma P., Guoping Z., Woon O.H. A UWB based localization system for indoor robot navigation. Proceedings of the IEEE International Conference Ultra-Wideband.

[18] Baala O., Zheng Y., Caminada A. The impact of AP placement in WLAN-based indoor positioning system. Proceedings of the 8th International Conference on Networks.

[19] Mahfouz M.R., Kuhn M.J., To G., Fathy A.E. (2009). Integration of UWB and wireless pressure mapping in surgical navigation. IEEE Trans. Microw. Theory Tech..

[20] Abdulrahman A., Abdulmalik A.S., Mansour A., Ahmad A., Suheer A.H., Mai A.A., Hend A.K. (2016). Ultra wide band indoor positioning technologies: Analysis and recent advances. Sensors.

[21] Sobhani B., Zwick T., Chiani M. (2016). Target TOA association with the Hough Transform in UWB radars. IEEE Trans. Aerosp. Electron. Syst..

[22] Gong X., Zhang J., Fang J. (2015). A modified nonlinear two-filter smoothing for high-precision airborne integrated GPS and inertial navigation. IEEE Trans. Instrum. Meas..

[23] STM32 32-Bit Arm Cortex MCUs. https://www.st.com/en/microcontrollers-microprocessors/stm32-32-bit-arm-cortex-mcus.html.

[24] Razor_imu_9dof. http://wiki.ros.org/razor_imu_9dof.

[25] Kais M., Morin S., De La Fortelle A., Laugier C. Geometrical model to drive vision systems with error propagation. Proceedings of the ICARCV 2004 Control, Automation, Robotics and Vision Conference.

[26] Sabatini A.M. (2006). Quaternion-based extended Kalman filter for determining orientation by inertial and magnetic sensing. IEEE Trans. Biomed. Eng..

[27] Yun X., Lizarraga M., Bachmann E.R., McGhee R.B. An improved quaternion-based Kalman filter for real-time tracking of rigid body orientation. Proceedings of the 2003 IEEE/RSJ International Conference on Intelligent Robots and Systems.

[28] Zeng Y., Kirkland J.W., Anderson J.F., Leftin L.J., Briske R.W. (2011). Methods and Systems for Implementing an Iterated Extended Kalman Filter within a Navigation System. U.S. Patent.

[29] Tiano A., Sutton R., Lozowicki A., Naeem W. (2007). Observer Kalman filter identification of an autonomous underwater vehicle. Control Eng. Pract..

